# How does social network in patent provide changes in the Chinese manufacturing firm market value?

**DOI:** 10.1016/j.heliyon.2023.e14358

**Published:** 2023-03-09

**Authors:** Kai Luo, Shutter Zor

**Affiliations:** School of Accountancy, Wuhan Textile University, Wuhan, Hubei, China

**Keywords:** Patent thickets, Patent cooperation network, Firm market value, China, Patent maintenance period

## Abstract

Patent thickets create external resource constraints that reduce the market value of enterprises. In the process of technological innovation, it is no longer possible to obtain competitive advantage by relying on individual resource endowment alone. Patent cooperation is considered an important institutional element in social network relations, through which enterprises can integrate external innovation resources to counteract their shortcomings and obtain competitive advantages. In this study, 419 electronic equipment manufacturing enterprises in China between 2009 and 2019 are used as the research objects, and panel data include the financial data of enterprises, the number of patent citations, and the number of patents jointly applied for by enterprises. Based on the patent fragmentation index, social network analysis is used to construct a patent cooperation network. Using UCINET and Gephi tools, the structure of corporate patent thicket networks and their evolutionary characteristics can be obtained. A panel regression model is used to empirically test the social network effect and the effect of patent thickets on the market value of firms in the context of patent cooperation. The findings show that patent thickets can discount the market value of enterprises. However, if a firm is at the center of patent cooperation, it can obtain more innovation resources. If it is at the intermediary of cooperation, it can absorb external knowledge and technology and occupy a richer structural hole, which can positively regulate the negative effect of the patent thicket. In addition, the negative effects of patent thickets are weaker for firms with more than five years of patent maintenance compared to those with less. Our study has important implications for improving the efficiency of patent cooperation and helping governments and firms to effectively cope with patent thickets.

## 1 Introduction

With the reform of China's patent law and the bias of China's “pro-patent” policy, the number of patents in China has shown a trend of “patent blowout” [1]. For example, from 2012 to 2021, the China National Intellectual Property Administration granted a total of 3.953 million invention patents, with an average annual growth rate of 13.8%. Altogether, 156,000 high-tech enterprises in China have 1.339 million valid invention patents, accounting for 64.2% of the valid invention patents of domestic enterprises. In this context, it can easily develop into a “patent thicket”, which is seen as a manifestation of the constraint of innovation resources due to the explosive increase of patents [[2], [3], [4]]. Patent thickets are the result of a combination of endogenous continuous innovation to a certain extent, and exogenous patent policy promotion, and they are formed in each technology field where there are a large number of complementary patents that belong to many patent holders [5]. The overlapping effect of patent rights increases patent licensing fees in a fragmented patent market, which is referred to as the licensing fee stacking problem [6]. Prior research identified the blocking effect of patent thickets on biomedical technologies and the theory of patent “anti-tragedy of the commons” was subsequently proposed [7,8]. In order to obtain licenses for core patents, companies need to obtain licenses for many peripheral patents, increasing the cost of investment [9]. The flood of patent applications and licenses has led to the creation of many patents without scientific experimentation, only logical deduction, which has created an obstacle to the commercialization of core technologies [10]. Resultantly, patent thickets are bound to have a negative impact on the market value of companies. Therefore, based on the Chinese context, this study analyzes the relationship between patent thickets and the market value of enterprises, and simultaneously explores an effective path for enterprises to cope with patent thickets. These research objectives have important implications for the innovation development and high-quality patent management of Chinese enterprises.

Existing studies have verified patent thicket density in specific technology areas based on the “fragmentation index” constructed from the Herfindahl index and the number of citation triangles of patentees [11,12]. Semantic analysis has been used instead of expert assessment to determine the evolution of patent thicket density in combination with patent citations [13]. Patent thickets in the field of new energy vehicles are diverse among countries, and each country has different policies to deal with them [14]. Existing literature has also focused on the impact of patent thickets on innovation performance [10,15,16]. It has been shown that patent thickets lead to an explosion in the number of patents, and thus the evolution of Non-Practicing Entities [17], which reduce the likelihood of patent countersuits and increase the cost of litigation [10,18]. Patent thickets also lead to “fragmentation of patent rights” along the complete technology chain [19]. Moreover, patent thickets allow overlapping patent rights, obligating firms to engage in repetitive innovation, thereby wasting research and development (R&D) resources [20,21]. Patent thickets increase the cost of innovation and patent commercialization and reduce the market value of firms [22]. Patents, as an output of innovation, are ultimately intended to help firms earn excess profits, and studies have shown that patent thickets lead to risks or expensive patent acquisition costs in the marketplace, reducing firm market value [23,24]. However, some studies have found that the negative effect of patent thickets on firm market value can be suppressed if firms can effectively acquire patent resources for their portfolios [20]. Studies have shown that social network relationships built through patent collaboration can help firms earn revenue [25,26], which is a different function from traditional R&D. Can firms use the unique function of the social network of patent collaboration to mitigate the negative effect of patent thickets on firm market value? As far as we are aware, no studies in the literature address this question.

Therefore, this study aims to fill the aforementioned research gap. Specifically, we seek to understand the impact of the “informal system” established by patent collaboration on the escape from innovation resource constraints [27], that is, social networks, on improving the market value of firms. We use a sample of Chinese listed companies in the electronic equipment manufacturing industry for the period 2009–2019 to analyze their market value, patent thicket density, and social network relationship data on corporate patent collaboration. Our contribution to the literature is twofold. First, through empirical analysis, we advance our understanding of the impact of patent thickets on the market value of Chinese firms. Although the impact of patents on firm performance has been studied [28,29], patents are mainly the product of firm R&D and are internal resources of firms [30]. The market value of a firm is not only affected by internal resources but also by external resource constraints such as patent thickets. Research on the impact of the patent thicket on the market value of firms is lacking, especially in the Chinese context. In China, high-tech enterprises, as an important part of the national innovation system, provide the necessary support for innovation-driven development. Technological innovation has an impact on the market value of enterprises through both the incentive of patent protection and the crowding-out effect of patent thickets, which are an inevitable defect of the patent system, as highlighted by Rai and Price [31]. Presently, China's patent development also happens to be at the stage of “patent blowout.” Therefore, it is important to avoid “patent mines” in enterprise technology innovation; improve the high-quality development and commercialization of enterprise patents; and identify the degree of the impact of patent thickets on the market value of Chinese enterprises.

Second, by constructing a patent cooperation network and combining it with the impact of patent thickets on enterprise market value, we strengthen our understanding of the latter (i.e., the impact of patent thickets on enterprise market value) under social network context factors. This innovative development should overcome resource constraints formed by the patent thicket. It is imperative to adopt effective governance mechanisms to mitigate the crowding-out effect of the patent thicket. Enterprises are constrained by patent thickets, and increasingly realize that they can no longer gain competitive advantages solely through their own resource endowments in the process of technological innovation. Furthermore, the social network relationship through patent cooperation can not only integrate external innovation resources to compensate for enterprises' shortcomings [32,33], but can also become a strategic tool to protect the weak firms. The social network relationship of patent cooperation is an important institutional element. If firms conduct R&D cooperation while putting themselves in a favorable position in the cooperation network, it is beneficial for them to acquire integrated internal and external resources, which reduce the asymmetry of information or the cost of technology use [34,35]. Entezarkheir [36] argues that firms acquire patent resources that can mitigate patent thickets' negative effects on firms' market value. China's patent protection system remains imperfect, and the role of patent cooperation—as the most important path for firms to acquire patent resources [37]—in the impact of patent thicket on the market value of firms has not been studied. Existing research considers patent resources as a whole, but firms have very different paths to accessing these patent resources [38], and their roles in the process of patent thickets influencing firm market value differ. This study posits that the network relationships formed by firms in patent cooperation, and the structural characteristics of firms in the network are important contextual factors that regulate the relationship between patent thickets and firms' market value. We provide new empirical evidence for the analysis of patent thickets and social network theory by comprehensively considering the relationship between patent cooperation networks formed by enterprises and the characteristics of node locations, as well as analyzing the moderating effect of enterprise patent cooperation networks in the patent thicket affecting enterprise market value. We also provide suggestions for governments and enterprises on how to cope with the patent thicket by improving the efficiency of patent cooperation networks.

The remainder of the paper is structured as follows. Section 2 provides a literature review and presents our research hypotheses; section 3 describes our sample selection principles, data sources and variable treatments, and research model; section 4 reports the results of our empirical study; and Section 5 presents the discussion. Section 6 summarizes the research and concludes the paper.

## 2 Literature review and hypotheses development

### 2.1 Patent thickets and firm market value

Patent protection with firm market value is a trade-off between the super marginal concept of monopoly distortion and increasing the number of available ideas through innovation [39]. Excessive granting of patent rights to inventors and the blurring of patent boundaries reduces the relative usage space and value of individual patents, which could affect firms’ returns. Patent thickets give firms ownership of patents but not usage rights in the technology chain, which runs counter to the idea of breaking down barriers to facilitate the flow of resources. As a result, when firms are constrained by patent thickets, their market value will be harmed to a certain extent.

Apart from studies on the relationship between patent thickets and firm market value, there has been research into the mechanism by which patent thickets affect firm market value in terms of patent-related costs [24,40]. Firms tend to shy away from investing in the R&D of technologies with severe patent thickets [5,41,42], and increase the growth of internal patents to obviate the use of the technologies of external patentees, incurring costs and reducing firm market value [20,22]. However, a high number of internal patents has an advantage in that it mitigates the patent thicket's adverse effect on firm market value [36]. Firms enter into patent transaction agreements with holders of complementary external patents because this helps to reduce their transaction costs and improve performance [22,43]. Therefore, firms' patenting behavior could affect their market value through an increase or decrease in patent costs [30,44].

At present, the mechanism of the impact of patent thickets on the market value of a firm is unclear and the existing research needs to be expanded. The market value of a firm reflects the present value of the firm's future cash flows as a result of investors' participation in the firm's evaluation [45]. The return to investors is the long-term performance of the firm, which can be constrained by external resources [40]. However, previous studies have primarily focused on the impact of patent thickets on firm innovation performance [46]. The impact of patent thickets on firm market value in the Chinese context has not been addressed. At present, Chinese enterprises rely on technological innovation to improve their competitiveness. The innovation process needs to be carried out through the “introduction-absorption-reinvention” model of new technological knowhow, or the “springboard model” of “following a certain path-leapfrogging-creating” to achieve the transition from imitator to innovator [47]. The “springboard model” facilitates the transition from imitator to innovator [47,48]. Thus, a harsh patent thicket environment hinders technology adoption and harms firms' incentives to innovate, inevitably lowering market value [16,24]. Based on this discussion, we propose our first hypothesis.

H1 Patent thickets have a negative effect on firm market value.

Next, we suggest that the effect of patent thickets on firm market value is different if firms have different patent maintenance durations. This refers to the actual time from the date of patent application, or the granting of a patent, to the date of invalidation, termination, revocation, or expiration of the patent [49]. Patent maintenance duration is one factor used to gauge the value and the quality of patents. However, Chinese firms generally do not maintain their patents for long periods of time [50]. Significant differences are found in the performance of firms with different patent maintenance periods [51], perhaps because the longer a firm maintains its patents, the better the quality of its patents, and the more it can obtain excess returns. In addition, according to the principle of market signal transmission, a firm's willingness to invest considerably in maintaining its patents indicates that its patents are strong; this information is favorable to the market, helps attract more investors, and increases the firm's future market value. In contrast, the maintenance of patents involves considerable transaction costs; if a firm's patents are not effectively commercialized and cannot bring sustainable monopolistic gains, it is reluctant to continue maintaining its patents, leading to greater uncertainty regarding a rise in firm market value [52]. Therefore, it is necessary to divide the study's research samples based on the length of patent maintenance.

### 2.2 Patent cooperation networks, patent thickets, and firm market value

A patent cooperation network refers to the social network relationship formed by two or more applicants who jointly apply for patents [53]. When patent rights are shared by multiple applicants, patent cooperation can be seen as a product of collaborative R&D [54]. Some scholars use patent cooperation networks to measure R&D cooperation, whereby patents are seen as the output of R&D; this reflects the specific content of the technology, its application, and the results of innovation, while revealing R&D cooperation performance and the path of innovation [55]. Social networks can help firms gain a competitive advantage and increase their firm market value [56,57]. According to resource-based theory, the competitive edge of firms lies not only in their resources but also in the network relationships that facilitate acquiring, allocating, and exchanging these resources [58]. Some scholars also consider network relationships as a form of inimitable social capital that helps with gaining access to unique resources and knowhow [59]. In contrast to traditional organizations that rely on “human connections” to obtain resources, social network relationships formed by patent cooperation have an important impact on firms’ access to patents and technological innovation [60]. Regarding electronic devices, for example, Zhongxing Telecommunication Equipment (ZTE) has signed patent cross-licensing agreements with several collaborators globally and cooperated with universities and research institutions when applying for patents.

Existing literature has yet to examine the role of patent cooperation networks in the relationship between patent thickets and firm market value. Open innovation theory suggests that organizations gradually shift from independent to collaborative innovation [61]. Some studies have shown that participation in an informal network facilitates knowledge sharing, exploits synergies, and promotes the flow of knowledge between organizations [62]. It is evident that social networks facilitate the formation of open innovation [63]. However, little existing literature has examined the importance of patent collaboration as an important open innovation model for firms to escape from external resource constraints and influence firm performance. Through patent cooperation, cooperative parties can effectively grasp, combine, and integrate the common technologies they possess, which contributes to improved business performance [53]. The main indicators to measure social networks are network density, degree of network centrality, structural holes, betweenness, and closeness to the center [57,64]. These metrics are adopted in this study to gauge patent cooperation network relationships.

Network density is the ratio of the number of edges present in the network to the upper limit of the number of edges that can be accommodated and can be used to characterize the density of interconnected edges between nodes in a network [25]. In high-density network structures, there is a larger number of strong ties, faster information transfer, and easier access to resources [65].

We posit that firms can obtain social capital through network relationships formed by patent cooperation. By embedding themselves into the network in an appropriate and suitable fashion, firms can focus their limited time and energy on identifying high-quality resources and avoid blindly searching for patents in external technology markets, eschewing over-dependence on external patent behaviors. High-density network linkages can help firms obtain valuable resources to easily form a win-win model of collaboration, which in turn facilitates a faster rise in firm market value [66]. If firms maintain an appropriate level of patent cooperation network density, they can reduce the negative impact of patent thickets on firm market value. Accordingly, we propose the following hypothesis.

H2 Network density has a positive moderating effect on the relationship between patent thickets and firm market value.

Network locales, considered key indicators in the social network analysis method, describe the structural characteristics of the network [67,68]. In a patent cooperation network, the positions occupied by a firm in the network lead to securing different knowledge resources that affect the company's profitability. Network centrality indicates how many targets an acting agent is connected to in a social network; a higher value indicates more members and, therefore, more power and influence. In addition, nodes with a high level of centrality are more likely to develop mutual trust relationships, shared norms, and common behavioral patterns with other nodes [69], which facilitate rapid spread of innovative knowledge and results.

Each firm is endowed with unique technological resources and capabilities. A firm's technological innovation behavior is embedded into the patent cooperation network, enabling it to acquire other complementary knowledge and technologies. By sharing these patents, the firm can reduce the cost of patent acquisition [53], thus eliminating the difficult situation where complementary patent resources are dispersed among different patentees, making it hard for firms to make efficient and integral use of the patents they need. This can indirectly help firms to increase their market value. In summary, when compared to firms in a marginal position of patent cooperation, those in the center position of the network have more resources at their disposal to break the technological barriers formed by the patent thicket and can identify and select information regarding the patents they need, thus reducing the degree of information asymmetry in the technology market caused by the patent thicket. Accordingly, we propose the following hypothesis.

H3 Network centrality has a positive moderating effect on the relationship between patent thickets and firm market value.

Patent cooperation establishes a network relationship that integrates technology, institution, and economics. The firm's position in the network determines whether it has better access to resources and information. Firms located in the center of the local network and occupying a rich variety of structural holes are found to have significant advantages in information acquisition [69], which is conducive to patent acquisition—the smaller the constraint for a firm, the greater its capability of patent identification and selection.

**Fig. 1:**
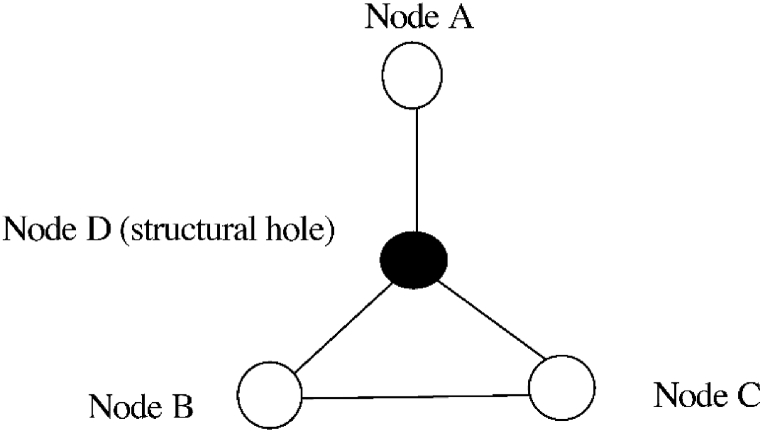
Illustration of a structural hole.

Accordingly, we believe that the more companies occupy the structural holes in the patent cooperation network, the more they can break the “resource curse,” that is, the patent thicket causing fragmentation of patent rights, thus alleviating its negative effect on their market value. In the process of coping with the patent thicket, firms should integrate and absorb internal and external patents to form their own comprehensive technology chain. Firms that occupy more structural holes in the cooperative network tend to play the role of intermediaries for organizations connected to them, improving the efficiency of knowledge spillovers and the ability to access information [70,71]. Organizations occupying structural holes can access heterogeneous, non-redundant, and timely knowledge and information from both ends of the structural hole, thus facilitating the recombination of this knowledge and technology into new iterations [72]. Firms in the intermediary bridge position often have the advantage of independent R&D with less constrained structural holes [72,73]. In addition, control advantages enable firms to gain greater bargaining power and more control over resources or outcomes through bridging connections in collaborative innovation; this allows them to effectively build strategic alliances and enact decisions in their favor [72,74]. Fig. 1 illustrates the structural hole.

The greater the variety of structural holes in the patent cooperation network, the more firms can promote knowledge sharing and technology. The formation of common technologies cultivated can facilitate better integration and reorganization of the cooperative partners' respective fragmented patented technologies, which is conducive to overcoming the negative effect of the patent thicket on firm market value. Through patent cooperation, inter-organizational communication is strengthened [54]. In a patent cooperation network, firms’ non-redundant contacts are reduced at the beginning and knowledge homogenization is increased. The cohesive nature of cooperation density reduces the complexity of innovation validation, which ensures that organizations not constrained by structural holes have access to more appropriate information resources relatively quickly [75]. Contrastingly, when firms acquire patents in an external patent thicket environment, patent cooperation networks reduce the cost of searching for non-redundant information, mitigate the risks to organizations, and help firms develop economies of scale. Accordingly, we propose the following hypothesis.

H4 Structural holes have a positive moderating effect on the relationship between patent thickets and firm market value.

Research suggests that firms in these intermediary positions can link two areas of dense relationships, thus bringing new information to these linked parties and allowing resources to flow through this new linkage [55]. Social network theory suggests that such intermediary positions tend to create asymmetries in network resources, increasing the degree of the dependence of firms connected to the network, which then gain higher status and more resources that are turned into competitive advantages. Many scholars believe that firms located in network intermediary positions show better performance than those in marginal positions [76].

Innovation is a continuous, cumulative investment process [14] that needs the support of many prior patents. Firms’ patent cooperation network relationships often serve as a useful bridge to improve understanding between cooperative partners and break through resource barriers. The intermediary bridging effect in social networks can help firms access resources easily and at low cost [77]. The “intermediating mechanism” in a patent cooperation network generates resource access paths that can be translated into economic benefits, increasing innovation opportunities and reducing costs [53]. Thus, the following hypothesis is proposed.

H5 Betweenness has a positive moderating effect on the relationship between patent thickets and firm market value.

In the patent cooperation network, firms close to the central belt can not only obtain a larger competitive edge, but also gain and utilize external complementary resources by identifying these from inter-firm relationships and the network [33]. The interaction between firms in the network can generate knowledge externalities in addition to systematic network effects, helping to improve the organizational and learning capacity of firms within the network, thus helping them to achieve sustained competitive advantages [73]. In the context of patent fragmentation, it is relatively difficult for any one firm to achieve product innovation and dominate the market independently. In addition, the closer a firm is to the center of the patent network the greater the economies of scale it can achieve. Areas close to the center in the patent cooperation network can be seen as relatively independent zones in which it is possible to synergistically integrate the technology chain in a rapid fashion, thus cultivating integrated expansion capabilities and economies of scale to help companies enhance their market value. Accordingly, we propose the following hypothesis:

H6 Closeness has a positive moderating effect on the relationship between patent thickets and firm market value.

**Fig. 2:**
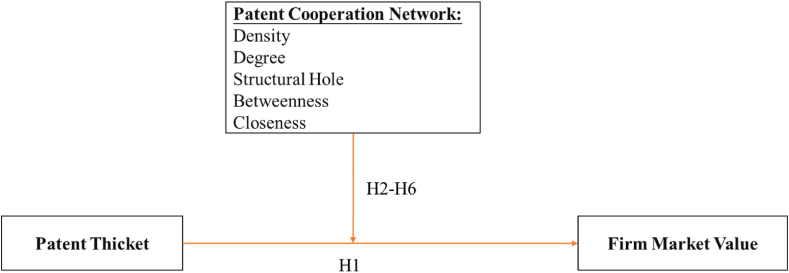
Conceptual model of analysis.

The conceptual framework of this study is presented in Fig. 2.

## 3 Data sources and research methods

### 3.1 Data sources

Our study sample was drawn from the China Stock Market & Accounting Research (CSMAR) database for the 10-year period 2009–2019. We first selected our samples from a limited range by targeting the electronic equipment manufacturing sector, under the secondary technical classification of the China Securities Regulatory Commission in 2012. We excluded companies penalized for unusual financial status, and those that had continuously made losses, eliminating companies that were delisted from the Shanghai Stock Exchange STAR Market, Main Board, ChiNext market, or SME board. We also excluded all companies from the financial and insurance sectors. Companies with incomplete/missing data were excluded during processing. The final sample came to 419 companies. In our panel data regression, we considered the inventions and patents of the sample firms, and the technical information included therein, attributes, technical fields, citations of the patents by other patent owners and the protection scope of the patent rights. We used a Python program to extract the patentees who jointly applied for patents from the sample firm's information, who were then matched as pairs, and the intersection of the two patentees was the number of jointly applied-for patents. We used the Derwent database for firms' patents data. We used the 419 selected companies to search for the desired corporate patents in the Derwent database using the name of the patentee as the search term and our selected time period, 2009–2019. We performed manual data cleaning on the raw data, merging patents of the same patentee and excluding homologous patents to ensure the accuracy of the company patent data. The core explanatory variables of this study are centered on patent thickets, and we investigated patent thickets by focusing on individual enterprises. Based on prior studies, we constructed a fragmentation index to study patent thickets at the individual firm level [12]. The fragmentation index was constructed based on the citation data of company patents to ensure that all patents of the 419 sample firms included patent citation data. We extracted the patent data of all the different patent owners cited by the company and built a patent citation database. To measure the density of the patent thicket, based on the fragmentation index, we calculated the number of patents per year for which citations were made by each company from different patentees, as well as the total number of patents cited by each company.

For financial data, namely firm market value, the explanatory variable in this study, we followed Yang et al. [78], and selected Tobin's Q to measure it. Firms' R&D investment data and the Herfindahl index (HHI) were also obtained from the CSMAR database. Since the data were sourced from different databases, Stata14.0 was used to perform a 1:1 matching for stock code and year. If such a matching was not possible, manual processing was performed through Excel. Ultimately, a total of 4609 observations were obtained for the period 2009–2019 for the 419 sample firms.

### 3.2 Method

We refer to the firm market value assessment model proposed in previous studies [36,[79], [80], [81]]. The empirical econometric model is described in Equation (1):

(1) $$\log\;q_{it}=a_{0}+b_{1}\;\log\;{\left(frag\right)}_{it}+\sum c_{i}Control_{it}+Year_{i}+a_{i}+\varepsilon$$

where the logarithm of Tobin's Q (log(q)) is the dependent variable. Equation (1) denotes the market value of firm i in year t; the logarithm of patent fragmentation index (log(frag)) denotes the patent fragmentation value of firm i in year t; the fragmentation index is constructed and taken in its logarithm to measure the patent thicket index (log(frag)) [12,36], as shown in Equation (2).

(2) $$frag_{it}=1-{\sum_{i\neq j}^{J}\left(\frac{cite_{ijt}}{cite_{it}}\right)}^{2}\text{,}$$

where cite_ij_ indicates that enterprise i cites the number of patents of patentee j. In equation (2), it represents the number of all patents cited by the enterprise. Each reference in the patent document is viewed as a complementary patent to the main patent. The smaller the value, the more complete the patent rights, the larger the value, and the more fragmented they are. The control variables, based on a previous study [81], the R&D stock (RDstock), patent stock (patstock), and patent citation stock (citestock) of the firsms are calculated. From this, the logarithm of R&D intensity (log (RDstock/TA)), the logarithm of patent density (log(patstock/RDstock)), and the logarithm of the degree of complementary patents (log(citestock/patstock)) of the firm are measured, as shown in Equation (3).

(3) $$Constock_{it}=\left(1-\delta \right){Con}_{i,t-1}+{Con}_{it}$$

Patstock is the number of patents granted in the previous period converted to present value plus the number of patents granted in the current year, based on which, RDstock and citestock can be calculated. TA is the total number of corporate assets, with the δ value taken as 15%. The Herfindahl index, in its logarithm form (log(hhi)), is used to measure market monopoly power and ε denotes the error term. In addition, year and individual dummy variables are considered in this model to control for their effects.

Equation (4) is extended to estimate the moderating role of the firm patent cooperation network in how patent thickets affect firm market value.

(4) $$\begin{array}{l} \log\;q_{it}-c_{0}+\delta_{1}\;\log\;{\left(frag\right)}_{it}+\beta_{1}\;\log\;{\left(\text{co}-\text{patent}\;\text{network}\right)}_{it}+\beta_{2}\;\log\;{\left(frag\right)}_{it}\times \\ \log\;{\left(\text{co}-\text{patent}\;\text{network}\right)}_{it}+\sum \gamma_{i}Control_{it}+Year_{i}+a_{i}+\varepsilon \end{array}$$

where the firm co-patent network is measured as the logarithm of network density (log(Den)), the logarithm of network degree (log(Deg)), structural holes (SH), the logarithm of betweenness (log(Bet)), and the logarithm of closeness (log(Close)). An interaction term is added to Equation (4); the log(frag) × log (co-patent network) interaction in Equation (9) is estimated by the parameters of the interaction term.

We designed the calculation formula of density (in equation (5)) based on Burt [74], and the density of the firm patent cooperation network is calculated with UCINET software; the logarithm of the density value is then taken as the density value of the firm patent cooperation network, as follows.

(5) $$Density_{i}=\frac{\sum S_{j}k}{2\left(N^{2}-N\right)}$$

where Density_i_ denotes the density of the patent cooperation network of the ith firm, S_j_k denotes the closeness of the relationship between node i and k in the patent cooperation network of the firm, and N denotes the size of the network.

#### 3.2.1 Network degree

Degree indicates the total number of cooperative patents in the patent cooperation network that are directly linked to the firm and is used to measure the degree of the firm's closeness to the network center. The larger the value, the higher the frequency of synthesis of the firm in the network, and the more likely that the firm is a core enterprise in the network. We design the formula of Equation (6) and use UCINET software to calculate the degree value of the firm patent cooperation network [32], and the logarithm of the degree value is then taken as the degree value of the firm patent cooperation network.

(6) $$Degree_{i}=\sum_{j}x_{ij}$$

where Degree_i_ denotes the centrality of the ith firm patent cooperation network, and x_ij_ denotes the number of nodes i directly connected to j.

#### 3.2.2 Structural holes

Based on the previous study [75], we design Equation (7) to measure the structural hole indicator of the firm and use UCINET software to calculate the structural hole value of the firm and the firm patent cooperation network.

(7) $$Structural\;hole_{i}=\frac{\sum_{j}\left(1-\sum_{q}p_{iq}m_{jq}\right)}{C_{j}}$$

where structural hole_i_ denotes the structural hole of the ith firm. P_iq_ denotes the ratio of connections between firm i and partner q, m_jq_ denotes the ratio that firm i is simultaneously connected with j and q, and C_j_ denotes the total number of partners in the network.

#### 3.2.3 Betweenness

In the patent cooperation network, a firm has a high intermediary centrality if it is on the path that many nodes must pass through [27]. We design the formula of equation (8) and use UCINET software to calculate the betweenness value of the firm; we take its logarithm as the betweenness value of the firm patent cooperation network.

(8) $$Betweenness_{i}=\sum_{k<k}\frac{G_{jk}\left(n_{i}\right)}{G_{jk}}$$

where betweenness_i_ denotes the centrality of the ith firm, G_jk_ denotes the number of paths that are the shortest between node j and node k, and G_jk_(n_i_) denotes the number of the shortest paths between node j and node k that pass-through node i.

#### 3.2.4 Closeness

This value reflects the firm's independence in acquiring patent resources. The smaller the shortest distance between the firm and other nodes in the network, the larger the closeness value [55]. Based on Equation (9) using UCINET software, the closeness value of the firm is calculated, and its logarithm is taken as the closeness value of the firm patent cooperation network.

(9) $$Closeness_{i}=\frac{n-1}{\sum d\left(i,j\right)}$$

where closeness_i_ denotes the ith firm's degree of closeness to the center, n denotes the total number of nodes within the network in which node i is located, and d(i,j) denotes the length of the shortest path from node i to node j. The definitions of all variables and their symbolic representations are presented in Table 1.

**Table 1:** Definitions of relevant variables.

Type	Variables	Symbol	Definitions of variables	Previous studies involving variables
Explained variable	Firm market value (Tobin's Q)	logq	Used for measuring the firm market value; its logarithm is taken in this study.	Entezarkheir [36] examined the impact of patent thickets on the market value of firms using Tobin's Q, a US manufacturing firm
Explanatory variable	Patent thicket (fragmentation index)	log(frag)	The logarithm of the fragmentation index is taken in this study	Ziedonis [12] and Entezarkheir [36] measured patent thicket density using the patent fragmentation index
Moderating variables	Density	log(Den)	The density of firms' patent cooperation network; the logarithm of density is taken in this study.	Burt [74] measured network Density in social networks with Density
	Degree	log(Deg)	The degree of centrality of the firm in the patent cooperation network; the logarithm of the degree value is taken in this study.	Hwang [32] uses Degree to measure node centrality in a patent cooperation network and identify which organizations are core collaborators
	Structural holes	SH	Firms' structural hole value in the patent cooperation network	Guo et al. [75] found that the more abundant network node structural holes in the R&D network in the ICT field, the more beneficial to network expansion
	Betweenness	log(Bet)	The intermediary centrality value of firms in the patent cooperation network; the logarithm of betweenness is taken in this study.	Xia et al. [27] showed that Chinese universities and research institutions played an intermediary role in China's COVID-19 patent cooperation network
	Closeness	log(Close)	The degree value of the firm's closeness to the center in the patent cooperation network; the logarithm of the closeness is taken in this study.	Kwon et al. [55] is used to measure the closeness of patent cooperation and network location
Control variables	R&D Intensity (RDstock/TA)	log(RDstock/TA)	The ratio of firm R&D stock to total assets; the log form is taken in this study.	The control variables are based on Hall et al. [81] and Entezarkheir [36]
	Degree of patent complementarity (citestock/patstock)	log(citestock/patstock)	The ratio of the stock of patent citations to the stock of patent assets of a firm; the log form is taken in this study.	
	Patent density (patstock/RDstock)	log(patstock/RDstock)	The ratio of firms' patent asset stock to R&D stock; the log form is taken in this study.	
	Herfindahl Index (HHI)	log(HHI)	A measurement of market monopoly power; the log form is taken in this study.	

We first use Equation (1) to examine H1, that is, the relationship between patent thickets and firm market value. A negative regression coefficient indicates that patent thickets harm firm market value. We use Equation (4) to examine H2–H6, which focus on the moderating role of the patent cooperation network in the relationship between patent thickets and firm market value. Then, to observe the differences in the relationship between the patent thicket, patent cooperation network, and firm market value under different contexts of patent maintenance period, we divide the sample into firms with a patent maintenance period of more than 5 years and less than 5 years, before running regressions on these two groups separately.

## 4 Results

### 4.1 Descriptive statistics and analysis

The descriptive statistics of the sample variables are shown in Table 2. The mean of log(frag) is −0.087, while that of firm market value is 0.764. The descriptive characteristics are similar to data for variables in previous studies [36]. The Hausman test is performed for each model separately, and the results show that the p-value of each model test is less than 0.01; therefore, the random effect is rejected, and the fixed effect model is used.

**Table 2:** Descriptive statistics for all variables.

Variables	Mean	SD	Median	min	max
log(q)	0.764	0.463	0.513	−0.270	3.066
log(frag)	−0.087	0.104	−0.060	−0.693	−0.000
log(Den)	0.342	5.323	0.000	−5.099	87.498
log(Deg)	4.307	0.565	4.605	1.079	4.605
SH	0.787	0.282	1.000	0.000	1.531
log(Bet)	3.531	1.165	3.912	−1.532	4.605
log(Close)	4.142	0.827	4.605	0.000	4.605
log(RDstock/TA)	−1.506	1.031	−1.430	−29.239	3.981
log(citestock/patstock)	8.602	16.844	1.553	−4.706	83.357
log(patstock/RDstock)	−21.622	14.179	−15.793	−78.479	11.774
log(HHI)	−2.960	0.290	−2.981	−3.411	−2.518

### 4.2 Illustrating the patent thicket network using ZTE

**Fig. 3:**
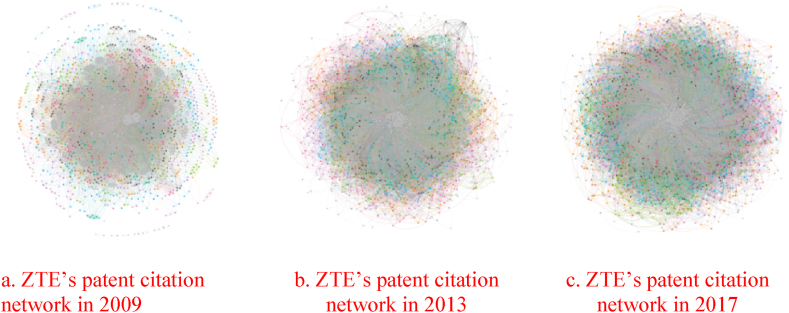
The patent citation network of ZTE. The gray nodes represent ZTE, while the colored nodes represent patentees cited in the company's patents. As time passes, the number of stray nodes shrinks and the firm citation relationship tightens, indicating that the company relies more on prior patents and more and more complementary patents emerge. A patent thicket trend is obvious.

**Fig. 4:**
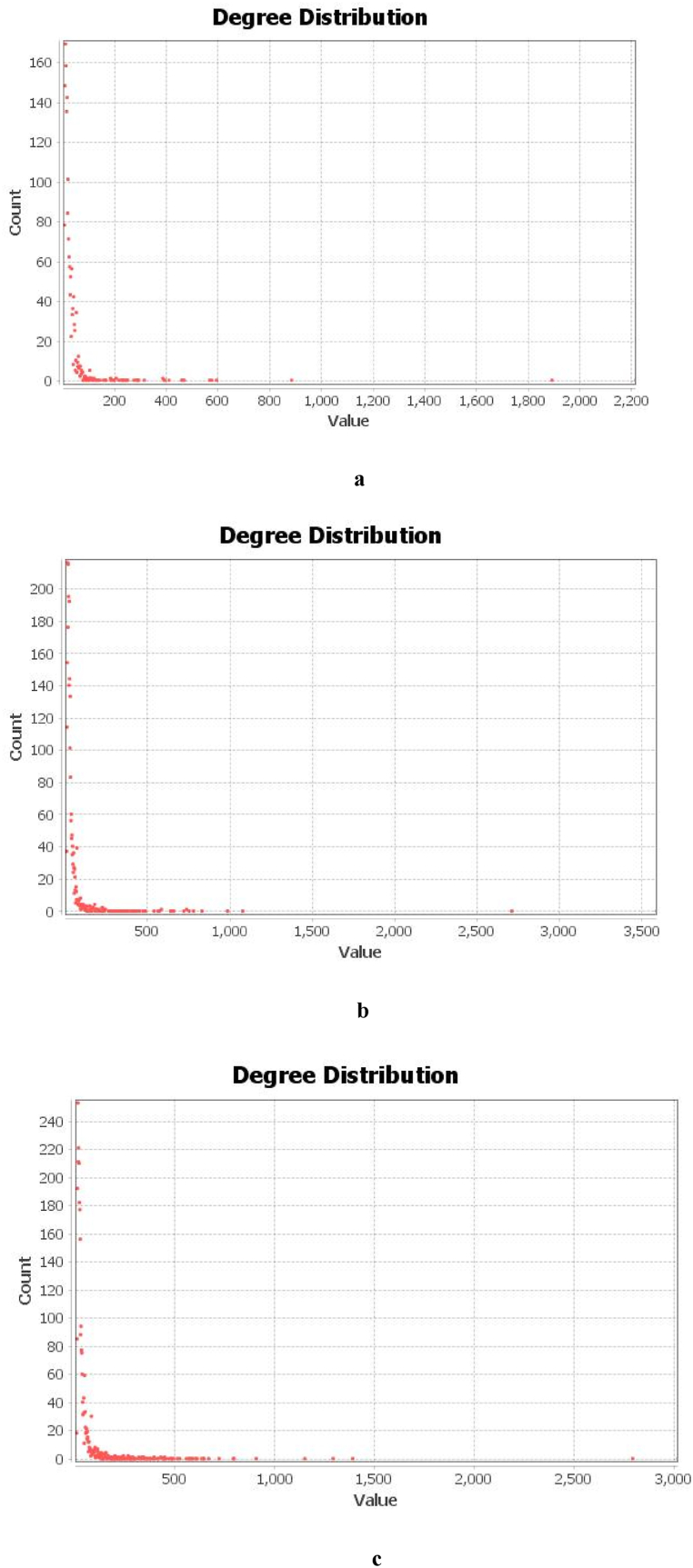
(a) The degree of network centrality of ZTE's patent citations in 2009 (average degree: 14.279). (b) The degree of network centrality of ZTE's patent citations in 2013 (average degree: 18.606). (c). The degree of network centrality of ZTE's patent citations in 2017 (average degree: 20.445). The figures shows that the network centrality degree increases over time, indicating that the company requires more complementary patents. The patent thicket is exacerbated by increasingly complex technology and evident patent fragmentation.

With carefully selected firm patent citation data, we used telecommunications company ZTE as an example to design a patent thicket network based on the corporate citations of different patent owners and the patent data of these owners. A specific analysis is conducted. First, we use python to extract the names and patent numbers of the patent owners cited by the firms based on their patent data in 2009, 2013, and 2017, before constructing a patent citation network matrix for each patent owner. Second, prior research indicates that the more patent rights and patents cited by a patentee, the more complementary patents the firm needs and the more serious the fragmentation of patent rights [15]. We import the constructed patent citation network into Gephi software and described the network graph of the patent thicket to calculate the centrality of the network, as shown in Fig. 3, Fig. 4c.

### 4.3 Regression results

Table 3 shows the results of the fixed effects panel regression. First, the fragmentation index has a negative impact on firm market value (see Model 2 β = −1.384, p < 0.01), which supports H1. Second, Table 4 shows the difference in the effect of patent thicket on firm market value in varying contexts of patent maintenance periods. We conduct fixed effects panel regressions for the sample of firms with patent maintenance periods of more than 5 years (tay>5) and less than 5 years (tay<5), and find that the fragmentation index of the sample group with tay>5 has a less negative effect on firm market value than with tay<5 —Model 3 β = −1.115, p < 0.01 and Model 4 β = −3.379, p < 0.01, respectively. This indicates that the longer the patent maintenance period, the better the quality and value of the patent, the more the firm can overcome market frictions caused by the patent thicket, and the stronger the firm's willingness to convert the patent, which could effectively mitigate the negative effect on firm market value.

**Table 3:** Panel regression results.

	Fixed effect model (FE)		Random effect model (RE)
Variables	logq	logq	Logq
	(1)	(2)	(3)
log(frag)		−1.384***	−1.200***
		(-4.92)	(-5.15)
log(RDstock/TA)	0.048**	0.048**	0.001
	(2.52)	(2.52)	(0.18)
log(citestock/patstock)	−0.017***	−0.017***	−0.018***
	(-4.48)	(-4.48)	(-5.22)
log(patstock/RDstock)	−0.011***	−0.011***	−0.013***
	(-2.65)	(-2.65)	(-3.15)
log(HHI)	−0.244***	−0.244***	−0.229***
	(-6.17)	(-6.17)	(-6.62)
Constant	0.031	0.031	−0.013
	(0.23)	(0.23)	(-0.10)
Firm Fixed Effect	Yes	Yes	No
Year FE	Yes	Yes	Yes
Observations	4609	4609	4609
Hausman Test	Prob > chi2 = 0.000		

Note: *^,^ **^,^ *** indicate significance at the 10%, 5%, and 1% levels, respectively. As shown in the table, the p value of the Hausman test result = 0, which is less than 0.1. The original hypothesis is accepted, and the fixed effect model should be used.

**Table 4:** Regression results broken down by group under different patent maintenance periods.

Variables	Logq		Logq	
	(3)		(4)	
	tay>5	tay>5	tay<5	tay<5
log(frag)		−1.115***		−3.379***
		(-4.47)		(-2.95)
log(RDstock/TA)	−0.040***	−0.040***	−0.028	0.132**
	(-3.31)	(-3.31)	(-0.75)	(2.48)
log(citestock/patstock)	−0.062***	−0.062***	0.167***	−0.032
	(-5.11)	(-5.11)	(2.63)	(-0.71)
log(patstock/RDstock)	−0.035***	−0.035***	−0.004	−0.025
	(-3.14)	(-3.14)	(-0.27)	(-1.35)
log(HHI)	−0.194***	−0.194***	0.087	−0.904***
	(−5.15)	(−5.15)	(0.44)	(-4.16)
Constant	−0.122	−0.122	0.997	−2.087***
	(−0.46)	(−0.46)	(1.60)	(−3.08)
Firm Fixed Effect	Yes	Yes	Yes	Yes
Year FE	Yes	Yes	Yes	Yes
Observations	2293	2293	292	292

Note: *^,^ **^,^ *** indicate significance at the 10%, 5%, and 1% levels, respectively.

**Fig. 5:**
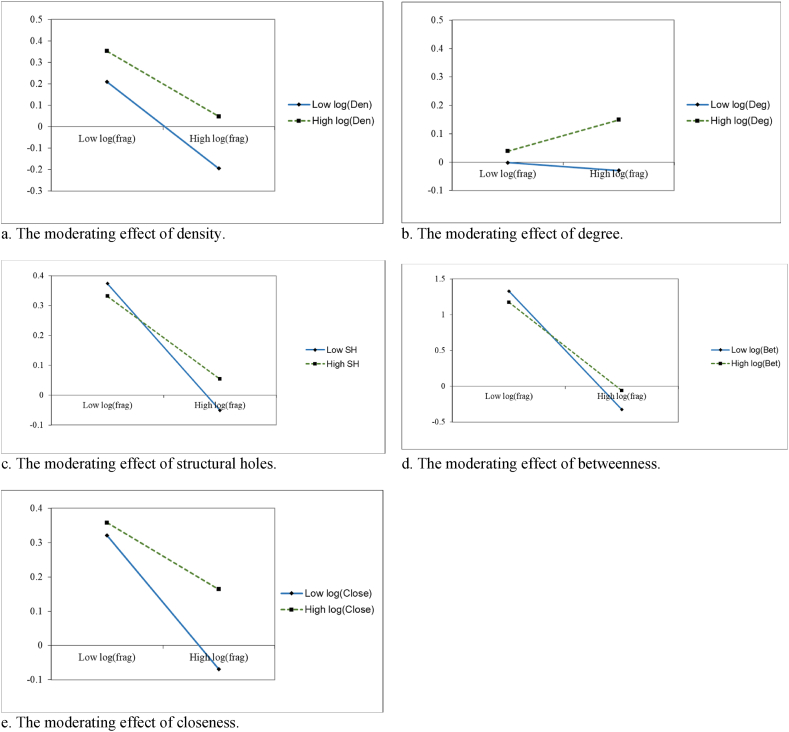
Moderating effects of the patent cooperation network.

Table 5 shows the regression results of the moderating effect of the patent cooperation network, and Models (5)–(9) show the negative effect of the fragmentation index on firm market value in all cases. Table 5 shows the results of regression coefficients of different patent cooperation networks on firm market value. In Models (5)–(9), the regression coefficients of the different networks are positive and significant, meaning the firm is in a favorable position in the patent cooperation network and maintains a close cooperative relationship with its partners to gain a competitive advantage and enhance its market value. Meanwhile, the regression coefficients of the interaction terms of different patent cooperation networks and log(frag) in Models (5)–(9) are all positive and significant, indicating that a firm's active participation in patent cooperation could effectively suppress the negative impact of patent thickets on its market value. Therefore, H2–H6 are supported. Fig. 5a–e shows the moderating effect in more detail, indicating that when the level of patent cooperation network is higher, the negative slope becomes flat or even positive.

**Table 5:** The moderating effect of the patent cooperation network.

Variables	Logq	logq	logq	logq	logq
	(5)	(6)	(7)	(8)	(9)
log(frag)	−1.718***	−2.342*	−2.678***	−10.004***	−3.758***
	(-3.37)	(-1.77)	(-2.98)	(-4.37)	(-2.74)
log(Den)	0.022***				
	(4.58)				
log(frag) × log(Den)	0.045*				
	(1.90)				
log(Deg)		0.148***			
		(3.25)			
log(frag) × log(Deg)		0.590**			
		(2.10)			
SH			0.164***		
			(3.01)		
log(frag) × SH			1.255*		
			(1.70)		
log(Bet)				0.099***	
				(2.86)	
log(frag) × log(Bet)				0.866**	
				(2.05)	
log(Close)					0.131***
					(4.54)
log(frag) × log(Close)					0.569**
					(2.24)
log(RDstock/TA)	0.043***	0.057**	0.063***	0.123***	0.059**
	(5.03)	(2.32)	(5.52)	(3.10)	(2.35)
log(citestock/patstock)	−0.016***	−0.016***	−0.016***	−0.027***	−0.016***
	(-4.32)	(-3.48)	(-4.33)	(-3.76)	(-3.37)
log(patstock/RDstock)	−0.010**	−0.010*	−0.010**	−0.023***	−0.009*
	(-2.37)	(-1.93)	(-2.40)	(-2.87)	(-1.76)
log(HHI)	−0.267***	−0.244***	−0.251***	−0.247***	−0.246***
	(-5.41)	(-4.81)	(-4.86)	(-3.08)	(-4.85)
Constant	−0.052	−0.581**	−0.099	−0.423	−0.471**
	(-0.32)	(-2.24)	(-0.57)	(-1.33)	(-2.27)
Firm Fixed Effect	Yes	Yes	Yes	Yes	Yes
Year FE	Yes	Yes	Yes	Yes	Yes
Observations	1626	1526	1529	644	1525

**Fig. 6:**
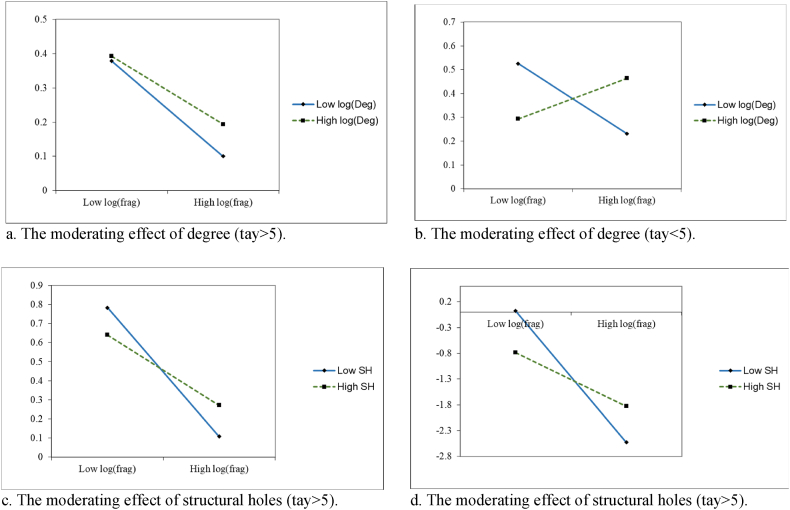
Moderating effects of the patent cooperation network under different patent maintenance periods.

Table 6 shows the regression results of the moderating effects of the patent cooperation network after grouping the samples by different patent maintenance periods. Models (10), (13), and (14) show that the moderating effects of log(Den), log(Bet), and log(Close) are not significant, that is, the moderating effects of the patent cooperation network indicators are not significantly different in different periods. This implies that if firms are not located in favorable patent cooperation positions, even if they hold proprietary patents for a longer period of time and have higher patent quality, they will be unable to effectively obtain resources and integrate their own technologies into the cooperation network to mitigate the negative effect of patent thickets on firm market value. Models (11) and (12) show that the regression coefficients of log(Deg), SH, and log(frag) interaction terms of tay>5 and tay<5 sample groups are positive and significant (i.e., Model 11 β = 0.339, p < 0.1; β = 1.973, p < 0.1, and Model 12 β = 1.427, p < 0.1; β = 6.994, p < 0.05). The regression coefficients of the above interaction terms for the tay>5 group are smaller than tay<5, that is, if a firm's maintenance time is shorter for its proprietary patents, it needs to engage in more patent cooperation, occupy a favorable position in the network, and increase cooperation with partners to obtain more patent resources. It is also necessary to consider occupying more structural holes, filtering out redundant resources, obtaining and integrating internal and external heterogeneous resources, and reducing the cost associated with resources to overcome the negative effect of a patent thicket effectively. We plot Fig. 6a–d to show more precisely the difference in the moderating effect under different patent maintenance periods. Thus, when SH is at a higher level, log(Deg) shows a negative slope in the tay<5 sample group and is flatter than in the tay>5 group. This indicates that firms with shorter patent maintenance periods can reduce patent-related costs and mitigate the negative effect of a patent thicket on firm market value if they are at the heart of the patent collaboration or located in an area with a rich variety of structural holes. We used Chow [82] tests to examine the variability of our regressions grouped by patent maintenance periods. The patent maintenance period is set as a dummy variable, with a value of 1 if the period is greater than 5 years (tay = 1) and is then interacted with log(frag). The test results are shown in Table 7. The interaction coefficients of tay and log(frag) in Models (3), (11), and (12) are significant (Model 3 β = −0.381, p < 0.1, Model 11 β = 11.202, p < 0.05, and Model 12 β = 4.574, p < 0.1). This means we can test for between-group differences in the effects of patent thickets on firm market value and the moderating effects of the patent cooperation network according to different groups of patent maintenance periods, which validates our grouping method.

**Table 6:** Moderating effects of the patent cooperation network under different patent maintenance periods.

Variables	logq		logq		Logq		logq		logq	
	(10)		(11)		(12)		(13)		(14)	
	tay>5	tay<5	tay>5	tay<5	tay>5	tay<5	tay>5	tay<5	tay>5	tay<5
log(frag)	−1.160**	1.438	−2.605**	−8.795*	−2.026**	−6.269**	−0.454	−5.153***	−1.328	−5.257*
	(-2.11)	(0.66)	(-2.34)	(-1.80)	(-2.09)	(-2.14)	(-0.41)	(-3.24)	(-1.64)	(-1.84)
log(Den)	0.012**	−0.044								
	(2.57)	(-0.82)								
log(frag) × log(Den)	0.009	−0.285								
	(0.41)	(-0.26)								
log(Deg)			0.077**	0.172*						
			(2.11)	(1.81)						
log(frag) × log(Deg)			0.339*	1.973*						
			(1.79)	(1.82)						
SH					0.134*	0.556**				
					(1.92)	(2.27)				
log(frag) × SH					1.427*	6.994**				
					(1.86)	(2.30)				
log(Bet)							0.034	0.105***		
							(1.09)	(2.65)		
log(frag) × log(Bet)							0.199	1.226*		
							(0.66)	(1.77)		
log(Close)									0.073***	0.148**
									(2.67)	(2.31)
log(frag) × log(Close)									0.335*	1.157*
									(1.82)	(1.80)
Control	Yes	Yes	Yes	Yes	Yes	Yes	Yes	Yes	Yes	Yes
Constant	0.236	1.079	−0.165	−0.388	0.278	−1.843***	0.182	0.000	−8.792***	0.000
	(0.68)	(1.39)	(-0.70)	(-0.68)	(0.75)	(-2.75)	(0.67)	(0.00)	(-3.89)	(0.00)
Firm Fixed Effect	Yes	Yes	Yes	Yes	Yes	Yes	Yes	Yes	Yes	Yes
Year FE	Yes	Yes	Yes	Yes	Yes	Yes	Yes	Yes	Yes	Yes
Observations	1428	198	1342	187	1344	189	559	98	1405	187

**Table 7:** Chow tests on the moderating effect of the patent cooperation network under different patent maintenance periods.

Variables	logq	logq	logq	Logq	logq	logq
	(3)	(10)	(11)	(12)	(13)	(14)
	tay = 1	tay = 1	tay = 1	tay = 1	tay = 1	tay = 1
log(frag)*dummy(tay = 1)	−0.381*	−0.176	11.202**	4.574*	2.482	2.436
	(-1.86)	(-0.53)	(2.41)	(1.65)	(1.45)	(0.61)
Constant	−10.855***	0.571	−1.135***	−0.229	−0.045	−0.237
	(-9.00)	(1.58)	(-2.85)	(-1.21)	(-0.14)	(-0.95)
Firm Fixed Effect	Yes	Yes	Yes	Yes	Yes	Yes
Year FE	Yes	Yes	Yes	Yes	Yes	Yes
Observations	4609	1626	1526	1597	644	1525

### 4.4 Robustness tests

Nonlinear regression (NLS) was used in the robustness tests for our main findings [12,36]. The control variables in the models of this study were first adjusted based on Equation (8) and then tested using NLS. The regression results in Table 8 demonstrate that the regression coefficient of log(frag) is negative and significant (Model 16 β = −0.236, p < 0.1), which supports the main hypothesis.

**Table 8:** Robustness tests.

Variables	(15)	(16)
logq		
log(frag)		−0.236*
		(-1.86)
RDstock/TA	0.188***	0.188***
	(4.25)	(4.25)
citestock/patstock	0.012**	0.013***
	(2.52)	(2.63)
patstock/RDstock	−4.99e-06***	−5.00e-06***
	(-3.74)	(-3.74)
log(HHI)	−0.435***	−0.454***
	(-5.71)	(-5.91)
Constant	−0.438*	−0.520**
	(-1.80)	(-2.10)
R^2^	0.049	0.051

In this study, patent fragmentation index(log(frag)) and patent asset(log(SumPatent)) interaction are lagged by one period. The explained variable log(q) and the explanatory variable log(frag) are lagged by one period for robustness considerations of variable substitution, and then quantile regression is performed. The results are shown in Table 9. In each quantile, the coefficient of log(frag) lagged one period is significantly negative, and the coefficient in the [0.2–0.6] quantile interval first increases, peaks at the 0.6 quantile, and then decreases, further supporting the research hypothesis of this study.

**Table 9:** Dynamicity test.

	Dependent variable: log(q)_t-1_				
Variables	20% quantile	40% quantile	50% quantile	60% quantile	80% quantile
log(frag) _t-1_	−1.007**	−1.557***	−1.629***	−1.765***	−1.470***
	(-2.46)	(-8.59)	(-6.45)	(-6.19)	(-3.87)
log(frag)_t-1_ × log(SumPatent)_t-1_	0.387***	0.591***	0.668***	0.576***	0.387***
	(3.35)	(8.48)	(5.45)	(5.19)	(3.35)
log(RDstock/TA)	0.059**	0.052**	0.030	0.008	0.019
	(2.48)	(2.39)	(1.41)	(0.41)	(1.31)
log(citestock/patstock)	−0.040***	−0.025*	−0.020	0.005	0.025*
	(-3.56)	(-1.93)	(-1.28)	(0.36)	(1.76)
log(patstock/RDstock)	−0.033*	−0.042*	−0.035*	−0.034	−0.030*
	(-1.69)	(-1.94)	(-1.89)	(-1.49)	(-1.81)
log(HHI)	0.044	−0.022	−0.017	−0.116**	−0.231***
	(0.78)	(-0.28)	(-0.24)	(-2.37)	(-3.55)
Constant	0.313	0.246	0.452	0.284	0.347
	(1.07)	(0.56)	(1.06)	(0.64)	(1.06)
Pseudo R^2^	0.036	0.034	0.030	0.033	0.033

## 5 Discussion

Patent thickets can discount firm market value [20]. Currently, to our knowledge, there is little literature on which factors positively moderate the negative effect of patent thickets on firm market value. In this study, 419 electronic equipment manufacturing firms in China from 2009 to 2019 were used as the study population. The patent thicket density was then measured based on the patent dispersion index, and social network analysis was used to construct a patent cooperation network and measure the network density, network degree, structural holes, betweenness, and closeness of firms in the network. The influence mechanism of patent thickets on the market value of enterprises in the Chinese context was empirically examined. This study expands the research on the relationship between patent thickets and enterprise market value by revealing the network mechanism of path dependence of patent cooperation and its moderating effect on the relationship between patent thickets and enterprise market value. As no study, as far as we are aware, has investigated the exogenous mechanisms that influence the relationship between patent thickets and firm market value, we attempted to fill the gap by developing a multidimensional framework that combines external environmental stimuli and strategic orientation. Our three major findings are as follows.

First, patent thickets have a negative effect on firm market value; this is consistent with previous studies which affirm a hindering effect of patent thickets on the increase in firm market value [36]. In addition, our study found that for every one-unit change in patent thicket density, firm market value decreases by 1.384. In the U.S. manufacturing sector, for every one-unit increase in patent thicket density, firm market value decreases by 0.066, perhaps because patent maintenance periods and quality are greater in the U.S. than in China. Subject to the patent thicket, firms manage to reduce the negative effects with strong patent assets. Contrastingly, Chinese companies are subject to more serious negative effects because of poorer patent maintenance periods and patent quality. This study, in comparison with previous research, improves on two aspects: (1) existing literature lacks empirical analyses of the effect of patent thickets on firm market value in China; our study provides such an analysis. (2) Previous studies, based on full-sample analyses, showed that the negative effect of patent thickets on foreign firm market value was less pronounced than that of Chinese firms [36] but did not examine the “unbalanced effect” of a patent thicket on firm market value under sample differences. Because the quality and value of patents in China are different and uneven compared to those in the U.S. [83], the effect of a patent thicket on firm market value in China may be “unbalanced.” Therefore, sample differences can conceptually and theoretically play a role in the effect of a patent thicket on firm market value. This study classifies the sample firms according to the length of patent maintenance, enriching research on how firms can deal with patent thickets in a targeted manner.

Second, the patent cooperation network has a moderating effect. According to resource-based theory, the effective acquisition and scientific allocation of heterogeneous resources can improve one's own performance and competitive advantage [84]. However, it ignores the difference of the effective path of resource acquisition. Enterprises are often in a passive position due to resource constraints and inevitably subject to situational constraints. Therefore, traditional resource-based theory cannot fully explain the contextual factors that influence the relationship between patent thickets and firm market value. Our findings confirm the view that increased firm R&D investment is ineffective in getting firms out of resource constraints from patent thickets [20]. Previous research has argued that firms can only cope with patent thickets by expanding their proprietary patent resources [36]. In contrast, we argue that a combination of endogenous and exogenous mechanisms is necessary to effectively respond to patent thickets. However, exogenous mechanisms (network relationships formed by firms' patent cooperation) play an important moderating role in changing the direction in which patent thickets affect firm market value. For example, previous studies have shown that firms' technological innovation behavior is embedded in patent cooperation networks, enabling firms to share patents and reduce patent acquisition costs [53]; firms at the center of the network can obtain the opportunity to define the norms of the industry or network, increasing their competitiveness [66]; and innovation agents occupying structural hole positions can effectively cooperate. In addition, structural hole-rich firms can have a significant advantage in information access [69,77]. Table 5 shows that the coefficient of the interaction term of the patent dispersion index with network centrality is 0.590 and significant at 5%, and the coefficient of the interaction term with structural holes is 1.255 and significant at 10%. These results indicate that the more a company is in the central zone of cooperation or occupies more structural holes in the patent cooperation network, the more beneficial it is to concentrate patent resources and reduce the cost of patent acquisition, thus overcoming the negative impact of patent thickets on the market value of the company. Our findings further enrich the abovementioned patent cooperation and social network studies. Additionally, this study deepens our theoretical understanding of how firm patent cooperation interacts with patent thickets. Research on social network theory emphasizes the need for synergy between internal and external factors [54]. However, how external factors influence the relationship between a patent thicket and firm market value is still unclear. This study attempts to fill this gap by integrating social network theory and resource-based theory into a theoretical framework and investigating the moderating mechanism from the perspective of patent cooperation. The results show that the network relationship formed by firms' patent cooperation and their position in the network are important contextual factors that regulate this relationship.

Third, the moderating effects of some patent cooperation network variables differ significantly under different patent maintenance periods. Table 6 shows that the adjustment effects of network density, betweenness, and closeness are not significant. Therefore, if the firm is not located in the center of the patent cooperation network (e.g., in the intermediary zone and close to the center), whether it maintains its proprietary patents has no significant impact on the network's moderating effects on the relationship between a patent thicket and firm market value. This may be because firms located at nodes at the edge of the network are more intent on cooperating and accessing resources, and undue growth in their proprietary patents could incur additional costs and the ensuing redundancy of resources would not be conducive to cooperation. Contrariwise, the interaction coefficients of the patent dispersion index with network degree and structural holes are positive and significant in both the sample groups with more and less than 5 years of patent maintenance. This indicates that, compared with firms with longer patent maintenance time, firms with shorter patent maintenance time need to rely on the patent cooperation network and share patents of partners. This enables firms to reduce the cost of information and negotiation with patent owners in the external market and increase the bargaining power of patent transactions, thus creating synergistic effects. These enterprises will strive to occupy a favorable position in the cooperation, increase cooperation with their partners, and acquire more patent resources. Simultaneously, they will occupy more structural holes, filter out redundant resources, acquire and integrate internal and external heterogeneous resources, and reduce the costs associated with resources. They can play a leading and bridging role in cooperation, establish a strong cooperative relationship, integrate internal and external resources, and effectively mitigate the negative impact of the patent thicket. Our study bridges the gap in the research on the differences in the impact of patent collaboration networks regulating patent thickets on firms' market value in different contexts. It further expands the study of patent thicket governance mechanisms and provides important insights for governments and firms to govern patent thickets. In addition, Resource-based theory suggests that firms' access to resources helps them improve their competitiveness [84], but often overlooks the differences in resource use and contextual factors. We argue that the duration of firm patent maintenance is an important contextual factor that influences how firms can modulate the negative effects of a patent thicket on firm market value using patent cooperation. Under different firm patent maintenance periods, we found no difference in the moderating effects of network density, betweenness, and closeness for firms at the edge, or in the intermediate zone, of the patent cooperation network. This indicates that firms can only passively absorb external knowledge and technology, and that an undue growth of proprietary patent assets will generate resource redundancy and unnecessary costs. Conversely, if the firm is at the heart of the cooperative network or occupies a richer structural hole, the longer the firm maintains its patents, the more capital it has to dominate the cooperation, integrate internal and external resources, promote its ability to cope with patent thickets, and mitigate the negative impact on its market value. Thus, our findings contribute to the expansion of research on the mechanism of the effect of patent thickets on firm market value and the combination of social network and patent cooperation, and the framework of social network and resource-based theories as well.

## 6 Conclusions and limitations

### 6.1 Conclusions

This study empirically analyzed the influence mechanism of patent thickets on the market value of enterprises and the moderating effect of patent cooperation. We found a negative effect of the patent thicket on firm market value, which is particularly evident in Chinese firms. Our study shows that for every 1% increase in the density of the patent thicket, firm market value is diminished by 1.384. Previous studies have found that patent thickets have negative effects on the nano- and pharmaceutical industries [9,85]. However, no relevant study has been conducted with a sample of Chinese firm data. Our findings have important implications for the government and enterprises regarding the management of patent thickets. The government should encourage enterprises to effectively concentrate on the required complementary patents and carry out systematic and integrated innovation of products and technologies, formulate a science and technology policy that is conducive to diversified technology models, shift from the previous “pro-patent” policy to one that focuses on high-quality patent development, raise the threshold of patent examination to avoid producing too many “junk” patents, and focus on developing policies to foster high-quality patents. Enterprises should focus on arranging patents in key technology fields in the industrial chain and try to maintain control of these patents themselves to form a relatively complete technology industrial chain, so that the coverage of patent rights of individual enterprises is relatively complete.

Furthermore, we not only verified whether Chinese firms face the same resource constraints from patent thickets as U.S. firms but also considered the differences in the role of the patent thicket on firm market value under different contextual factors. We subdivided the sample according to the length of patent maintenance, examined the effects of a patent thicket for different samples, and found that firms with longer patent maintenance periods are subject to weaker negative effects of patent thickets than those with shorter patent maintenance. We suggest this may be because firms investing resources to maintain patents are more likely to reach agreements with outside parties on patent deals, or innovate independently. This could reduce associated costs and mitigate the negative effect of a patent thicket on firm market value. This novel contribution could help improve firms' understanding of the patent thicket. Previous studies have only explored the moderating effect of patent thickets on firm market value from endogenous factors of the firm. Some studies have found that the more patents a firm's R&D inputs and outputs have, the greater the negative effect of patent thickets on firm market value is mitigated [20]. The patent cooperation network is an important path for firms to obtain patent resources [60]. Patent cooperation not only reduces the cost of searching for the required patents in external markets but also the cost of patent licensing or patent negotiation; it mitigates patent risks, overcomes external diseconomies, and improves firms' innovation efficiency. Resultantly firms can then effectively integrate internal and external complementary patents to alleviate the negative effect of a patent thicket on their market value. Therefore, our study fills the abovementioned gap. Simultaneously, our study also finds an effective path for governments and enterprises to improve patent cooperation to achieve a positive response to the patent thicket. Governments should actively play a guiding role by designating more relevant science and technology policies on promoting patent cooperation, especially industry-university-research cooperation, so that the cooperating enterprises can acquire more heterogeneous resources and enrich their own patent portfolios. Governments should focus on supporting leading enterprises and embedding them in the patent cooperation network, so that they can occupy the core position of the network or the structural hole-rich zone, effectively integrating internal and external technical resources and helping enterprises to cope with patent thickets. Enterprises, in addition to legal application channels, should also focus on participating in patent cooperation and strive to position themselves in the core of the network or the intermediary zone, to reduce the cost of paying extra for patents, obtaining more patents for subsequent innovation and market competition, and improving their innovation status. Enterprises can appropriately introduce prior dispute resolution mechanisms in the process of patent cooperation, and negotiate the establishment of prior patent licenses, R&D cooperation results, distribution measures of revenue, and shared patents on patent risks such as infringement, product income, and patent commercialization arising from the cooperation. Enterprises should rely on technology-driven and market-pulling dual incentives to jointly cope with the patent thicket problem.

### 6.2 Limitations and future research

Like most research in this area, this study has some limitations. Its primary drawback is that only 419 enterprises were selected as the research sample, and there are limitations in the industry selection. Thus, more in-depth research is needed before our research findings can be generalized. Only Chinese electronic equipment manufacturing enterprises were explored, and the sample has not been extended to different high-tech fields to compare the differences of patent thicket influence effects among different industries. We use the values of network density, network degree, structural holes, betweenness and closeness to represent the degree of patent cooperation, without considering other patent cooperation factors and other exogenous moderating variables. We also use Tobin's Q as a proxy for enterprise market value, ignoring the impact of patent thickets on enterprise non-financial performance. For example, the study of the mechanism of the impact of patent thickets on firms' technology integration, product innovation performance, and patent commercialization. Previous studies also share this limitation.

By grouping the sample firms according to patent maintenance duration, we found differences in how the patent cooperation network moderates the negative effect of patent thickets on firm market value. There is little difference in how the duration of patent maintenance influences the way density, betweenness, and closeness moderate the relationship between patent thickets and firm market value. It is different, however, for centrality degree and structural holes, which need more research.

Finally, the study is limited in its use of a single industry, and further examination into the differences in the impact of patent thickets in other industries in China remains a future task. Efforts can also be made to study the differences in the impact of patent thickets in China in the biological, nano, or artificial intelligence fields. In addition, the transmission mechanism, or the factors through which patent thickets affect firm market value in China, have yet to be explored.

## Author contribution statement

Kai luo: Conceived and designed the experiments; Performed the experiments; Contributed reagents, materials, analysis tools or data; Wrote the paper. Shutter zor: Analyzed and interpreted the data.

## Funding statement

Professor kai luo was supported by National Natural Science Foundation of China [71902151]; the Humanities and Social Sciences Foundation, Ministry of Education of the People's Republic of China [18YJC630113]; the Humanities and Social Sciences Project of Hubei Provincial Education Department [19Q082].

## Data availability statement

Data associated with this study has been deposited at https://dataverse.harvard.edu/loginpage.xhtml?redirectPage=%2Fdataset.xhtml%3FownerId%3D1.

## Declaration of interest's statement

The authors declare no conflict of interest.
